# Healthcare professionals’ experiences of delivering palliative and end-of-life care in ethnically diverse communities: a qualitative study

**DOI:** 10.1186/s12904-026-02082-4

**Published:** 2026-04-01

**Authors:** Jodi Emma Wainwright, Erica Jane Cook, Catherine Haslam, Elaine Tolliday, Nasreen Ali, Mehrunisha Suleman, Emma Wilkinson, Gurch Randhawa

**Affiliations:** 1 Institute for Health and Wellbeing Research, University of Bedfordshire, Luton, England; 2 School of Psychology, University of Bedfordshire, Luton, England; 3 Keech Hospice Care, Luton, England; 4 Ethox Centre, University of Oxford, Oxford, England; 5 Gulf University for Science and Technology, Hawally, Kuwait

**Keywords:** Palliative care, End of Life, Ethnic minorities, Health inequalities, Research partnership network, Community engagement

## Abstract

**Background:**

Persistent inequalities in palliative and end-of-life care continue to affect ethnically diverse populations in the UK, with disparities in access, engagement, and quality of care. This study explored the perspectives of health professionals and service providers on barriers and facilitators to equitable palliative and end-of-life care in England (Bedfordshire, Hertfordshire, and Milton Keynes). The research was embedded within the KEEPNET initiative, a Research Partnership Network aimed at co-producing solutions to local healthcare inequalities.

**Methods:**

A qualitative design was employed using semi-structured interviews with six purposively sampled professionals across statutory and third sector palliative and end-of-life care services in Bedfordshire, Hertfordshire, and Milton Keynes. Participants included clinicians, service managers, and hospice-based practitioners. Data were collected in March 2023 and analysed thematically using an inductive approach to identify key patterns in professional experiences and perceptions of delivering care to ethnically diverse communities.

**Results:**

Three overarching themes were identified: (1) access to services (2), uptake of services, and (3) experiences of engaging with services. Barriers included language differences, mistrust of statutory services, cultural misconceptions around palliative and end-of-life care and limited outreach effectiveness. Facilitators included compassionate, individualised care; trust-building through culturally sensitive communication; and community engagement through faith and local leaders. Structural challenges such as workforce shortages and limited weekend provision were also reported. The Research Partnership Network model supported dialogue and capacity building between professionals and communities.

**Conclusion:**

Culturally competent, community-embedded approaches are essential to addressing disparities in palliative and end-of-life care. Research Partnership Networks offer a collaborative framework for developing responsive and inclusive care models that align with the lived realities of ethnically diverse populations. To ensure equitable care at the end of life, investment in cultural training, community partnerships, and co-produced service development is required.

**Supplementary Information:**

The online version contains supplementary material available at 10.1186/s12904-026-02082-4.

## Background

Providing timely and effective palliative and end-of-life care (PEoLC) is recognised as a critical quality standard for patients with incurable illnesses [1]. The UK *Better Endings* report [2] underscores that individuals receiving specialist PEoLC benefit from improved symptom management and are more likely to die at home, which can enhance the quality of their end-of-life experience [3]. However, despite the increasing ethnic diversity in the UK where over 25% of the population identify as non-White British, minority ethnic groups remain under-represented in PEoLC services [2, 4, 5]. This under-representation is associated with poorer symptom control, poorer satisfaction with services, and a higher likelihood of dying in hospital settings [5, 6].

Reducing such disparities has the potential to improve holistic care, ensure greater alignment with patient and family preferences, and enhance overall quality of life [7, 8]. Both national and international frameworks, including the NHS Long Term Plan, prioritise equitable, personalised, and accessible PEoLC for all populations [9–13]. Yet persistent barriers remain. These include negative perceptions of palliative care, limited awareness of services, cultural preferences around illness and death, language barriers, and experiences of discrimination [5, 14, 15]. Furthermore, many healthcare professionals report a lack of confidence and preparedness in delivering culturally sensitive care [16, 17].

Although our fieldwork was undertaken in England, inequities in access to palliative and end-of-life care are well documented internationally. Recent work since 2020 has foregrounded an ‘equity turn’ in palliative care, emphasising structural determinants, culturally responsive practice, and community partnership [18, 19]. We acknowledge related international work but do not attempt cross system comparison here.

To address these challenges, research must play a central role in understanding and responding to the needs of underserved communities. Strengthening research capacity within ethnically diverse populations is essential [20]. Research partnerships offer a promising approach by uniting stakeholders around shared objectives, fostering trust, and co-developing interventions that are culturally relevant and contextually appropriate [21]. Building local research infrastructure can generate impactful, community-driven evidence to inform policies and practices, ultimately helping to reduce inequalities in PEoLC [20].

This study focuses on England. For international readers, we briefly clarify the role of Research Partnership Networks (RPNs) as collaborative frameworks that unite researchers, healthcare professionals, policymakers, community representatives, and other relevant stakeholders to co-produce knowledge, design interventions, and implement solutions to address pressing societal issues [22, 23]. These networks aim to bridge the gap between research, practice, and policy by ensuring that academic findings are not only disseminated but are also applied in meaningful ways that can directly improve the lives of individuals and communities [24, 25]. In healthcare, RPNs are particularly valuable in addressing complex, multifaceted challenges, such as inequalities in PEoLC [26].

RPNs operate on the principle that the best solutions emerge from the collective expertise of diverse stakeholders [27]. Rather than working in isolation, researchers, healthcare providers, and community members collaborate to identify issues, define research priorities, and co-create strategies that are tailored to the specific needs of the populations they serve [25, 28]. This inclusive and participatory approach is particularly important in areas of healthcare where inequalities persist and where the effectiveness of interventions is often shaped by a range of social, cultural, and economic factors [29].

In the context of palliative and end of life care, Research Partnership Networks play a critical role in addressing disparities affecting people from disadvantaged backgrounds [30], defined here as those experiencing socioeconomic deprivation such as low income or insecure housing, limited English proficiency and health literacy, migration related barriers, and restricted access to services. These inequalities can manifest in numerous ways, including differences in access to timely, high-quality care, cultural sensitivity of services, and the degree to which healthcare providers understand and meet the unique needs of patients and families from diverse backgrounds [31]. Ethnic minorities, individuals from low socioeconomic groups, and those living in rural areas are often disproportionately affected by these barriers, which can result in poorer experiences of care and worse health outcomes [29].

One of the key strengths of RPNs in this field is their ability to facilitate collaboration between researchers and practitioners from various disciplines to design interventions that are both evidence-based and culturally competent [32]. By incorporating the perspectives of patients, families, and community organisations into the research process, RPNs ensure that care models are not only scientifically sound but also responsive to the values, preferences, and beliefs of the communities they aim to serve [22]. This ensures that interventions are relevant, practical, and sensitive to the diverse needs of individuals at the end of life [26].

Beyond improving the quality of care, RPNs also help to address systemic barriers that contribute to health inequalities in PEoLC [24]. These networks allow for the identification of structural issues such as the lack of culturally appropriate training for healthcare providers, limited access to palliative care services in certain geographic areas, and the underrepresentation of minority voices in healthcare policymaking [30]. By raising awareness of these issues and working collaboratively to find solutions, RPNs can contribute to systemic change and ensure that all individuals, regardless of their background, have access to equitable, person-centred care at the end of life [29].

The role of RPNs is particularly vital when it comes to ensuring that palliative care is inclusive and responsive to the needs of ethnic minority populations, who often face unique barriers to accessing care [32]. These barriers include cultural differences in attitudes toward death, religious beliefs regarding end-of-life care, language barriers, and historical mistrust of healthcare systems due to previous experiences of discrimination [31]. RPNs provide a platform for addressing these issues head-on, promoting dialogue between healthcare providers and ethnic communities, and ensuring that care models are tailored to the specific needs and preferences of these groups.

As the healthcare landscape continues to evolve, RPNs are playing an increasingly vital role in shaping policy, enhancing practice, and ensuring research reflects the diversity of the populations it serves [27]. This article explores professional perspectives on PEoLC within ethnically diverse communities, highlighting the complex, systemic factors that contribute to ongoing disparities. Drawing on findings from the KEEPNET initiative, an RPN designed to address inequities in PEoLC, we examine how collaborative, community-engaged approaches can generate more inclusive and culturally responsive care models. The following sections offer an in-depth analysis of professional insights into the barriers and enablers of equitable PEoLC. This contribution underscores the value of practitioner-led knowledge in shaping effective, community-centred responses to health inequalities at the end of life.

### KEEPNET research partnership network

The KEEch Research Partnership NETwork (KEEPNET) was developed to strengthen research capacity and capability in PEoLC across Bedfordshire, Hertfordshire, and Milton Keynes (BHMK). Funded by the National Institute for Health and Care Research (NIHR), KEEPNET represents the foundational phase of a wider programme of work aimed at tackling persistent health inequalities in PEoLC, particularly those experienced by ethnic minority communities in the region. The overarching research question guiding this initiative was: *How can access to PEoLC services be improved to reduce inequalities among the ethnic minority populations in Bedfordshire*,* Hertfordshire*,* and Milton Keynes?*

The KEEPNET project sought to build sustainable, inclusive research infrastructure by bringing together a diverse range of stakeholders, including academic researchers, clinicians, community leaders, hospice staff, and members of the public. By fostering collaborative relationships, the network aimed to generate locally grounded evidence and inform the design of culturally appropriate interventions that respond to the lived experiences and needs of underrepresented groups. In doing so, KEEPNET promoted a model of co-production that centres on equity, trust, and community relevance at every stage of the research process.

Activities within the KEEPNET initiative included the mapping of existing research activity in PEoLC across BHMK, identifying gaps in inclusion and representation, and conducting community engagement events to understand barriers to care. The project also involved capacity-building workshops for healthcare professionals and community members to support their involvement in future research projects. Ultimately, KEEPNET laid the groundwork for a more inclusive and responsive regional research ecosystem, helping to ensure that future studies in PEoLC reflect the diversity of the populations they intend to serve and address the structural barriers that perpetuate inequality [33].

## Methods

### Study design

A qualitative study was conducted using semi-structured interviews to examine how professionals across PEoLC services understand and respond to the needs of ethnic minority communities. The study was underpinned by an interpretivist qualitative descriptive orientation; analysis followed the Framework Method with inductive coding and constant comparison. An interview schedule was developed specifically for this study to guide discussions and ensure consistency across interviews while allowing flexibility for participants to share their experiences in depth (supplementary file 1). The interviews, which took place in March 2023, focused on participants’ experiences of service delivery, perceived barriers to access, and opportunities to enhance cultural responsiveness within care pathways. This approach was chosen to elicit rich, contextualised insights into the structural and interpersonal dynamics that shape inequalities in PEoLC.

Purposive sampling was used to recruit professionals from a range of settings ensuring representation from both statutory and third sector providers. The diversity of roles and service contexts enabled exploration of perspectives across different points in the care system, supporting a holistic understanding of how equity in end-of-life care might be improved.

Participants were approached by email with an information sheet; those who agreed were sent a Microsoft Teams link and a preferred time was arranged. All interviews were conducted by CH, a research assistant trained in qualitative methods; CH had no prior relationship with participants and outlined her role and interest before each interview. Interviews were conducted via Microsoft Teams; video/audio recorded with consent and transcribed verbatim. Participants joined from a range of private settings, including home and workplace locations, according to preference and availability. All invited participants consented and completed an interview; there were no withdrawals.

### Setting

The research was conducted in Bedfordshire, Hertfordshire, and Milton Keynes (BHMK), a region in Southeast England with a population of approximately 1.5 million projected to reach 2 million by 2035 [34]. The region exhibits significant demographic complexity, with an aging population trend and substantial ethnic diversity concentrated in urban centres.

Luton, in Bedfordshire, represents the most ethnically diverse and socioeconomically challenged urban area within BHMK. Census data [35] indicates 54.6% of Luton’s residents identify with minority ethnic groups, including established Pakistani, Bangladeshi, Indian, African-Caribbean, and Irish communities alongside newer migrants from Eastern Europe, Turkey, Afghanistan, and various African nations. Designated as a “Marmot Town,” Luton contains five wards (local electoral districts that form the basic administrative units of local government in England) among England’s 10% most deprived areas, with child poverty rates (29.6%) significantly exceeding the national average [36].

Bedford, Milton Keynes, and parts of Hertfordshire also demonstrate notable diversity and localized deprivation. Bedford town features significant Italian and Polish communities with two wards falling within the 20% most deprived nationally [37]. Milton Keynes, one of England’s fastest-growing urban areas, contains substantial Black African, Polish, and Indian populations alongside concentrated deprivation in several estates [38]. While Hertfordshire demonstrates greater overall affluence, Watford and parts of Stevenage contain significant pockets of deprivation [39].

These demographic patterns create distinct challenges for service provision, including language barriers, cultural factors affecting health behaviours, and persistent geographical health inequalities across the region [40].

### Participants

Health professionals and service providers working across the health and social care spectrum were recruited to take part in this study. Participants were drawn from a diverse range of PEoLC services across BHMK, including NHS Trusts, hospices, primary care settings, and community health services.

Participants were recruited purposively to reflect a range of professional roles and service settings relevant to palliative and end-of-life care in ethnically diverse communities. Recruitment was guided by Malterud et al.’s information power framework [41], with the aim of capturing sufficient depth and variation in perspectives. While the overall sample was modest, it was considered adequate given the focused study aim, the specificity of the participant group, and the quality of dialogue achieved. This approach aligns with accepted qualitative standards, where smaller, information-rich samples can yield meaningful insights into complex issues.

The sample included 5 women and 1 man, with ages ranging from approximately 30 to 60 years. Participants’ professional experience in palliative and end-of-life care varied from 2 to 30 years, however, time in current role was not reported. This diversity of roles, backgrounds, and experiences provided a broad perspective on the delivery of care in ethnically diverse communities. In addition to role, gender, and age range, we report aggregated ethnicity categories where feasible. Given the small sample and identifiable roles in a defined geography, we aggregate to protect anonymity.

Participants included an Advanced Nurse Practitioner based at a hospital trust in Milton Keynes, an Interim Lead Nurse for Reparative Care within Bedfordshire community health services, a GP surgery manager from a rural primary care setting, a Lecturer Practitioner working across clinical and academic hospice care, a clinical staff member from a regional hospice, and a Medical Director from a specialist hospice service. This cross-sectoral sample encompassed both clinical and managerial roles, enabling exploration of issues related to policy, service delivery, and direct patient care.

Participants were identified through professional contacts, the KEEPNET Research Partnership Network, and targeted outreach to ensure inclusion of underrepresented service areas. Their insights contributed to a holistic understanding of how PEoLC is currently delivered, the barriers and enablers to equitable access, and the extent to which services are equipped to meet the needs of ethnically diverse communities (Table 1).

**Table 1:** Participant details

Participant ID	Gender	Age range	Role	Ethnic category	Organisation Type
P1	F	40–50	Advanced Nurse Practitioner	White	Hospital Trust
P2	F	50–60	Interim Lead Nurse, Reparative Care	White	Community Health Services
P3	F	30–40	Clinical Staff Member	Asian	Regional Hospice
P4	F	40–50	GP Surgery Manager	White	GP Surgery
P5	M	50–60	Lecturer Practitioner	White	Hospice and Academic Setting
P6	F	40–50	Medical Director	White	Specialist Hospice

### Materials

Information and promotional materials about the study were distributed via email to relevant contacts identified through service mapping and the KEEPNET RPN. In addition to direct outreach, RPN members shared recruitment materials through their wider professional and community networks to enhance reach and representation. A total of six health professionals and service providers took part in the qualitative phase reported here, representing a range of PEoLC contexts across BHMK. Participants were drawn from hospital (1), community (2), and third sector (3) settings, and represented geographical areas including Milton Keynes [2], Bedfordshire [2], and Hertfordshire [2]. This diverse cross-section of roles and service types enabled a rich exploration of professional perspectives on access to PEoLC among ethnically diverse communities.

An interview topic guide was collaboratively developed by the multi-disciplinary research team, informed by literature and the KEEP-NET launch event discussions. The guide used open-ended questions to explore key research priorities, experiences of providing PEoLC to ethnic minority communities, perceptions of inequalities, communication and translation, planning for death, and approaches to faith and resource planning. Probes were used for further explanations when needed.

Participants were given a participant information sheet outlining the study’s nature and purpose, with written informed consent obtained through a consent form. A trained research assistant facilitated all interviews online via Microsoft Teams, lasting approximately 60 min each. Interviews were video/audio recorded with permission and stored securely. Participants could verify their transcripts, and video files were destroyed afterward. Pseudonyms were used to maintain confidentiality, and organisation names were removed from quotes.

### Data analysis

The interviews were analysed using the Framework Method [42] with an inductive approach. This involves a structured, transparent approach to identifying themes through a ‘matrix’ that highlights commonalities and differences in the data. Analysis proceeded in staged steps: familiarisation through repeated reading of transcripts; initial open coding on a subset of transcripts by two researchers (JW and EC); development of a working analytical framework through comparison and consolidation of codes; and application of this framework to the full dataset in NVivo 12. A working analytical framework was presented to the steering group for feedback and then refined before being applied consistently to all transcripts. The coded data were synthesized into a Framework Matrix with case nodes representing different PEoLC settings (hospitals, hospices, NHS community services) and coded nodes representing the themes. The interpretive phase involved constant comparison within and across cases, attention to negative or disconfirming evidence, and iterative memo writing to capture analytic decisions. To enhance credibility, a subset of transcripts was double coded independently, with differences resolved through discussion and consensus; an audit trail of codebook versions, memos, and framework iterations was maintained. Illustrative quotations were selected to represent typical and divergent views and are attributed by participant number, with demographic details aggregated where necessary to reduce the risk of deductive disclosure. Analysis was led by JW (EdD) and EC (PhD), female qualitative researchers with over 20 years of experience and postgraduate training in Higher Education, Psychology and Public Health, including prior work in palliative and end of life care and minority health; regular peer debriefs supported reflexive consideration of assumptions and positionality throughout.

## Findings

We present professionals’ perspectives and therefore report what participants perceived rather than service user accounts. We distinguish system issues that appeared generic from mechanisms that disproportionately affected minoritised communities (e.g. language and health literacy, mistrust linked to migration histories, documentation barriers) (Table 2).

**Table 2:** Themes, subthemes and illustrative quotes

Theme	Subtheme	Illustrative quotation (participant)
Access to services	Cultural sensitivity and barriers	“We need to ask the person in front of us what matters to them, not assume based on a tick-box or their surname.” — P2, Interim Lead Nurse, community health services
Access to services		“People don’t come forward early enough because they don’t know what the service is, or they’re suspicious — they think we’re going to push something on them.” — P1, Advanced Nurse Practitioner, hospital
Access to services	Language and interpreting	“Language can be a huge block. If someone doesn’t speak English and there’s no interpreter, we lose them before we even start.” — P2, Interim Lead Nurse, community health services
Access to services	Community engagement and awareness	“We work with mosques and churches to build relationships and share what we do.” — P2, Interim Lead Nurse, community health services
Access to services	Misconceptions about hospice care	“People think it’s just where you go to die — they don’t realise we offer emotional support, family care, and help earlier on in the illness.” — P3, Clinical staff, regional hospice
Access to services		“We still see a lot of fear around hospice care, and part of that is just not knowing what we actually do.” — P6, Medical Director, specialist hospice
Uptake of services	Communication and trust	“It’s about how we talk to people — not just what we say. We need to build trust from the first conversation.” — P2, Interim Lead Nurse, community health services
Uptake of services		“Words like ‘palliative’ or ‘end-of-life’ can be scary… We need to explain that it’s about comfort and support.” — P3, Clinical staff, regional hospice
Uptake of services	Mistrust of the system	“For some families, there’s a deep-rooted suspicion of the system — maybe from past experiences or from what they hear in the community.” — P1, Advanced Nurse Practitioner, hospital
Uptake of services	Communication under emotional/clinical load	“Even if someone speaks some English, the moment it’s emotional or medical, it becomes harder. We lose people at that point.” — P6, Medical Director, specialist hospice
Uptake of services	Community and faith partnerships	“We’ve worked with churches, mosques, and community centres — sometimes just showing up with tea and talking about what we do.” — P2, Interim Lead Nurse, community health services
Uptake of services	Familiar settings and sustained presence	“Holding sessions in familiar places makes the conversation feel less clinical, more human. It opens the door.” — P3, Clinical staff, regional hospice
Uptake of services	Tailoring and visibility of inclusion	“It’s not about parachuting in with a leaflet. It’s about being present, listening, and adapting.” — P3, Clinical staff, regional hospice
Uptake of services		“When people see themselves reflected in the service, they’re more likely to believe it’s for them.” — P2, Interim Lead Nurse, community health services
Experiences of engaging with services	Compassionate, person-centred care (hospice)	“In the hospice, we have time to sit with families, to ask what matters — not just medically, but personally and spiritually.” — P2, Interim Lead Nurse, community health services
Experiences of engaging with services		“People feel safe here. It’s not just about managing symptoms — it’s about dignity, rituals, family.” — P4, GP surgery manager, primary care
Experiences of engaging with services		“Hospice care often feels more human — it gives space for cultural and emotional needs.” — P5, Lecturer Practitioner, hospice/academic
Experiences of engaging with services	Resource and staffing constraints	“We’re doing our best, but we’re limited — we can’t offer seven-day services… evenings when things are toughest.” — P2, Interim Lead Nurse, community health services
Experiences of engaging with services		“The will is there, but without more staff and investment, it’s hard to meet everyone where they are.” — P6, Medical Director, specialist hospice
Experiences of engaging with services	Training and development	“We need to keep learning… You can’t assume — you need to ask, reflect, and adapt.” — P2, Interim Lead Nurse, community health services
Experiences of engaging with services	Communication and documentation across settings	“We have situations where someone’s wishes are known in one setting, but that doesn’t get communicated to the next team. That’s where things break down.” — P5, Lecturer Practitioner, hospice/academic

The analysis of interviews revealed three overarching themes that reflect professionals’ perspectives on the challenges and opportunities in delivering equitable PEoLC to ethnically diverse communities. These themes are interrelated and span the continuum of care, highlighting systemic, organisational, and interpersonal factors that influence outcomes for patients and families. The first theme, access to services, captures the structural and informational barriers that prevent individuals from entering the PEoLC system. The second theme, uptake of services, explores the factors that affect whether individuals choose to engage with PEoLC once they are aware of available options, including cultural perceptions, trust, and relevance of care. The third theme, experiences of engaging with services, addresses how care is received once accessed, with particular attention to communication, cultural sensitivity, and the adaptability of services to diverse needs. Together, these themes provide a nuanced understanding of how professionals perceive and navigate ethnic inequalities in PEoLC and point to areas for systemic improvement.

### Access to services

#### Cultural sensitivity and barriers

Across all interviews, participants emphasised the importance of cultural sensitivity as critical to improving access to PEoLC. The need for personalised, non-assumptive care was particularly stressed by professionals in community and hospice settings. As the Interim Lead Nurse in a community health service (P2) explained, *“We need to ask the person in front of us what matters to them*,* not assume based on a tick-box or their surname.”*

However, barriers such as language differences, cultural misunderstandings, and mistrust of statutory services persist. P1, an Advanced Nurse Practitioner based in a hospital, described, *“People don’t come forward early enough because they don’t know what the service is*,* or they’re suspicious — they think we’re going to push something on them.”* P2 also added, *“Language can be a huge block. If someone doesn’t speak English and there’s no interpreter*,* we lose them before we even start.”* These themes were reflected across the dataset, with widespread agreement that building trust and improving communication are vital to enhancing early engagement.

#### Community engagement and awareness

Participants saw community engagement as key to improving access, particularly when led by trusted community or faith figures. P2 described efforts to *“work with mosques and churches to build relationships and share what we do.”* However, outreach was not always successful. P6, a Medical Director at a specialist hospice, reflected, *“Some communities just don’t respond — we need to find different ways of reaching them that feel safe and familiar.”*

Several participants noted ongoing misconceptions about hospice care. P3, a clinical staff member from a regional hospice, explained, *“People think it’s just where you go to die — they don’t realise we offer emotional support*,* family care*,* and help earlier on in the illness.”* P6 similarly commented, *“We still see a lot of fear around hospice care*,* and part of that is just not knowing what we actually do.”* These findings reinforce the need for tailored, culturally relevant engagement and education.

Collectively, these findings underscore the need for more tailored and proactive engagement strategies that not only raise awareness but also build trust and clarify the supportive role of PEoLC services well before end-of-life stages.

### Uptake of services

#### Communication and trust

Clear, empathetic communication was repeatedly identified as a cornerstone of improving service uptake among ethnically diverse communities. Participants stressed that healthcare professionals must not only convey information clearly but also demonstrate cultural humility and emotional sensitivity when discussing PEoLC. P2 emphasised the importance of advanced communication skills, stating, *“It’s about how we talk to people — not just what we say. We need to build trust from the first conversation.”* This was echoed by P3, who highlighted the need to clarify complex or emotionally charged terminology: *“Words like ‘palliative’ or ‘end-of-life’ can be scary. For some families*,* they think it means we’ve given up. We need to explain that it’s about comfort and support.”*

Persistent mistrust of healthcare providers among some ethnic minority communities was seen as a major barrier to uptake. P1 observed that *“for some families*,* there’s a deep-rooted suspicion of the system — maybe from past experiences or from what they hear in the community.”* These concerns were compounded by language barriers, which were noted by P1, P2, and P6 as ongoing challenges. As P6 stated, *“Even if someone speaks some English*,* the moment it’s emotional or medical*,* it becomes harder. We lose people at that point.”* Participants agreed that improving uptake requires more than just translation—it calls for relationships built on trust, respect, and ongoing dialogue.

#### Community and faith group engagement

Engagement with community and faith groups was widely viewed as a promising approach to improving service uptake, particularly among populations historically underserved by PEoLC. Several participants described initiatives aimed at collaborating with trusted community figures and delivering education through culturally relevant forums. P2 shared, *“We’ve worked with churches*,* mosques*,* and community centres — sometimes just showing up with tea and talking about what we do.”* P3 added that holding information events in familiar settings *“makes the conversation feel less clinical*,* more human. It opens the door.”*

However, despite these efforts, uptake remains uneven. P6 reflected, *“We’ve done outreach*,* but the same people keep coming. It’s not reaching the groups we really need to reach.”* This sentiment was echoed by others, who noted that generic approaches often fail to resonate with the specific needs and concerns of marginalised communities. Instead, participants suggested that engagement strategies must be tailored, long-term, and co-designed with communities themselves. As P3 put it, *“It’s not about parachuting in with a leaflet. It’s about being present*,* listening*,* and adapting.”*

Several interviewees also stressed that making services more visibly inclusive—such as through diverse staffing, translated materials, or culturally appropriate spaces—could improve trust and participation. P2 concluded, *“When people see themselves reflected in the service*,* they’re more likely to believe it’s for them.”*

### Experiences of engaging with services

#### Compassionate, patient-centred care

Across all interviews, participants praised the compassionate, holistic care offered within hospice settings, often contrasting it with the more clinical and standardised approaches typically found in hospitals. Professionals described hospice care as being more attentive to individual needs, values, and cultural beliefs. P2 remarked, *“In the hospice*,* we have time to sit with families*,* to ask what matters — not just medically*,* but personally and spiritually.”* This emphasis on person-centredness was echoed by P4 a GP surgery manager and P5 a Lecturer Practitioner with hospice experience, who noted that patients and families often feel more respected and heard within these settings. P4 reflected, *“People feel safe here. It’s not just about managing symptoms — it’s about dignity*,* rituals*,* family.”* P5 added, *“Hospice care often feels more human — it gives space for cultural and emotional needs.”* This flexibility and attentiveness were viewed as essential to providing meaningful care, particularly for those from diverse cultural or religious backgrounds.

#### Resource and staffing challenges

Despite these strengths, participants also highlighted significant limitations in resources and workforce capacity. P2 and P6 expressed concern about the inability to extend services equitably, particularly to communities that are harder to reach or require more intensive support. P2 stated, *“We’re doing our best*,* but we’re limited — we can’t offer seven-day services*,* and some families need help over the weekend or evenings when things are toughest.”* P6 added that while demand for culturally appropriate care is growing, staffing levels and funding have not kept pace: *“The will is there*,* but without more staff and investment*,* it’s hard to meet everyone where they are.”* These limitations were seen as constraining the ability to deliver truly inclusive care.

#### Training and development

Training emerged as a recurring theme, with P2 emphasising the importance of continuous professional development focused on cultural competence and responsive care. As communities become increasingly diverse, staff must be equipped with the knowledge, confidence, and tools to support a wide range of beliefs, practices, and communication preferences. P2 shared, *“We need to keep learning. What works for one community doesn’t always work for another. You can’t assume — you need to ask*,* reflect*,* and adapt.”* Ongoing training was seen not only as a means to improve individual interactions but also to embed equity into organisational practice.

### Communication and documentation

Effective communication and documentation of patient preferences were also identified as essential to ensuring that care remains aligned with individuals’ values and choices throughout their journey. P4 and P5 pointed to challenges in information-sharing across teams, particularly during transitions between services. P5 explained, *“We have situations where someone’s wishes are known in one setting*,* but that doesn’t get communicated to the next team. That’s where things break down.”* Incomplete or inconsistent documentation can result in care that fails to reflect patient goals, leading to frustration for both families and professionals. Interviewees recommended improving systems for recording and sharing patient preferences across care settings, alongside strengthening inter-professional collaboration.

In summary, the interviews highlight a clear and consistent call for culturally responsive, person-centred care as fundamental to improving access, uptake, and experiences within palliative and end-of-life care services. Participants identified cultural sensitivity, clear communication, and meaningful community engagement as critical enablers of equitable care, particularly for ethnically diverse populations. At the same time, systemic barriers—including language challenges, mistrust, misconceptions about hospice care, and gaps in service provision—were seen to hinder early engagement and sustained participation. Addressing these barriers will require coordinated efforts to invest in workforce training, build stronger community partnerships, improve communication systems, and expand service accessibility. By embedding equity into everyday practice and policy, PEoLC services can better meet the complex and varied needs of the populations they serve.

## Discussion

This study explored the perspectives of health professionals and service providers on the barriers and enablers to accessing PEoLC among ethnically diverse communities in BHMK. The findings reinforce existing evidence that culturally sensitive, community-driven, and person-centred approaches are essential for addressing longstanding inequalities in PEoLC [29, 31]. Participants emphasised the importance of clear communication, trust-building, and cultural competence in improving access and the overall quality of care. *Cultural competence* refers to the knowledge, skills and organisational supports that enable effective work across cultures. These insights not only highlight persistent gaps in service provision but also point to the value of collaborative frameworks—such as RPNs—in co-producing practical, community-responsive solutions.Cultural sensitivity emerged as a foundational element of equitable PEoLC. *Cultural sensitivity* refers to the capacity to recognise, understand and respectfully respond to cultural differences— including values, beliefs, communication preferences and care practices— and to adapt interactions and care plans accordingly while avoiding stereotyping. Professionals across care settings stressed that individualised, non-assumptive care can enhance patient trust and engagement, particularly when cultural and religious values are acknowledged and respected. However, barriers persist, including language differences, misconceptions about palliative care, and long-standing mistrust of statutory health systems. These findings align with broader literature documenting the under-representation of ethnic minority populations in palliative care services and the associated disparities in access, symptom control, and patient outcomes [30, 31].

A central contribution of this study lies in its grounding within the KEEPNET initiative, a regional RPN designed to bring together academics, clinicians, community leaders, and service users to co-produce solutions to local health inequities. RPNs provide a collaborative infrastructure that moves beyond traditional research models by embedding patients and community voices directly into the research and intervention development process [22, 24]. The challenges identified in this study—such as community mistrust, underutilisation of services, and limited cultural competence—are not solely clinical issues, but systemic challenges shaped by historical, social, and institutional inequalities. RPNs offer a platform to address these through participatory and contextually tailored strategies [28].

Community engagement was consistently highlighted as a key approach to improving awareness and access. Participants described efforts to build trust and raise awareness through faith leaders and trusted community figures. While some of these initiatives were seen as effective, others were limited in their reach, particularly in engaging communities with lower levels of healthcare trust or awareness. These findings reinforce calls for sustained, culturally appropriate, and co-designed outreach strategies that reflect the diversity within and between ethnic groups [32].

Trust and communication were also viewed as core to improving service uptake. Participants noted how emotionally charged terminology—such as “palliative” or “end-of-life”—can evoke fear or confusion, particularly when not accompanied by adequate explanation or contextual understanding. These observations align with the wider literature and support the need for communication skills training and reflective practice for healthcare providers, especially when working with culturally diverse populations [27].

In addition to interpersonal challenges, structural issues such as limited staffing, constrained service models, and fragmented care pathways were noted as major obstacles to delivering culturally responsive care. Participants expressed the need to expand access beyond the current five-day model, improve weekend coverage, and enhance documentation and communication systems between care providers. These resource constraints are not unique to the BHMK region but reflect broader system-level issues affecting the equity and sustainability of PEoLC provision [26, 29].

Importantly, RPNs also serve as mechanisms for building research capacity within minoritised communities, enabling individuals and groups to shape the development of services that reflect their lived realities. The KEEPNET initiative facilitated dialogue between professionals and communities, supported the identification of locally relevant care priorities, and demonstrated the feasibility of co-designed approaches to service improvement [22, 25]. This type of collaborative model ensures that future interventions are both evidence-based and culturally grounded [32].

In summary, this study illustrates the complex interplay of interpersonal, institutional, and structural factors that shape access to palliative care for ethnically diverse populations. It also highlights the potential of Research Partnership Networks to support inclusive, sustainable solutions through equitable collaboration between researchers, practitioners, and communities. However, given the small and context specific sample, we are cautious about claims of transferability. These insights are most likely to resonate in comparable UK settings with similar population diversity and service configurations; their applicability to other regions or countries should be treated as provisional and explored through further research.

### Limitations

Findings arise from a small, context-specific sample in England and reflect professionals’ perceptions. This study involved a small sample of professionals, which may limit the transferability of findings to wider healthcare contexts. However, this is consistent with other qualitative research in palliative and end-of-life care, where in-depth insights are prioritised over generalisability. Similar studies, such as Fox et al. [43], which explored elements of effective dementia palliative care, and Allard et al. [44], which investigated expert recommendations for quality indicators, also used small, purposive samples to capture nuanced perspectives within complex care settings. As with those studies, the current findings offer valuable contributions to understanding the delivery of culturally responsive palliative and end-of-life care, while highlighting areas for further investigation with broader and more diverse participant groups. There is also a need for complementary studies capturing service users’ and carers’ experiences, particularly from minoritised communities.

## Conclusion

This study contributes to the growing body of evidence on the barriers and enablers to accessing PEoLC among ethnically diverse populations in England. Within this context, professionals identified intersecting interpersonal, institutional and structural mechanisms shaping access for ethnically diverse communities. RPNs may offer a promising platform for collaboration toward culturally sensitive practice; their impact requires further evaluation. Based on the perspectives of healthcare professionals and service providers, the findings highlight the persistent impact of cultural misunderstanding, language barriers, and mistrust in limiting access and engagement. At the same time, the study illustrates how community-based, culturally responsive approaches—supported by RPNs—can help address these challenges by building trust, fostering inclusion, and ensuring services reflect the needs and values of the populations they serve. The KEEPNET initiative demonstrates the value of collaborative, locally embedded frameworks in driving equitable innovation in PEoLC.

### Implications for policy and practice

- Cultural competence should be embedded across PEoLC training: Ongoing workforce development is needed to equip healthcare professionals with the knowledge and confidence to deliver culturally sensitive, inclusive care. This includes addressing language barriers, recognising diverse beliefs around end-of-life, and strengthening skills in compassionate communication [27, 32].
- Sustained community engagement must be prioritised: Health systems should invest in long-term, trust-building relationships with local communities, faith leaders, and grassroots organisations. Co-production of outreach strategies is essential for overcoming deep-rooted mistrust and misinformation [28].
- Address systemic service gaps to improve equity: Policymakers should address existing inequalities in service coverage, including gaps in weekend provision and community-based palliative care, particularly in underserved areas [26, 29].
- Enhance communication and continuity across care settings: Improved documentation and information-sharing between providers are vital to ensuring patient preferences, including cultural and spiritual needs, are upheld throughout the care journey.
- Invest in Research Partnership Networks to drive inclusive innovation: RPNs provide a robust mechanism for identifying local priorities, co-developing solutions, and embedding cultural relevance into policy and practice. Support for RPNs like KEEPNET can help shift research and service development towards more equitable and effective PEoLC models [22, 24, 25].

## Supplementary Information

Supplementary Material 1.

Supplementary Material 2.

## Data Availability

The data that support the findings of this study are available from Dr Erica Cook ( [erica.cook@beds.ac.uk] ) but restrictions apply to the availability of these data, which were used under license for the current study, and so are not publicly available. Data are however available from the authors upon reasonable request and with permission of Dr Erica Cook. No identifying images or other personal or clinical details of participants are included that compromise anonymity; therefore, consent to publish such details was not required.

## Abbreviations

BHMK: Bedfordshire, Hertfordshire, Milton Keynes
BLMK: Bedford, Luton, Milton Keynes
ICS: Integrated Care Service
NHS: National Health Service
NIHR: National Institute for Health and Care Research
PEoL: Palliative and End of Life Care
KEEP-NET: KEEch Research Partnership NETwork
RPN: Research Partnership Network
