# Supplementary material for: Healthcare professionals’ experiences of delivering palliative and end-of-life care in ethnically diverse communities: a qualitative study
Source: BMC Palliat Care. 2026 Apr 1;25:133. doi: 10.1186/s12904-026-02082-4 (PMC13169695; doi:10.1186/s12904-026-02082-4)
Supplement: Supplementary file 1 — Supplementary Material 1. [file 12904_2026_2082_MOESM1_ESM.docx]

## Supplementary file 1: Interview schedule

KEEPNet topic guide

| Background |
| --- |
| 1. Can you tell me a bit about what organisation you work for and your position within the organisation? 2. What is your role? (Responsibilities) 3. What organisations do you work closely with? |
|  |
| Research Network questions |
| Research experience   1. What has been your involvement in research?   Probe 1: Internal only, With third parties – charities, academics?  (Probe 2: specifically who, what, where, when, how)  1a If have been involved in research: What influenced you to be involved in research? What have been your experience(s) of being involved in research? What were the positives of taking part in research? What were the challenges? Did you feel your involvement was sufficient to contribute all you had to offer?  1b If not been involved: Why have you not been involved in research (Probe barriers: never asked, not appropriate, no time, lack of skills/lack of confidence in skills, no perceived need; lack of information and understanding/transparency of project; researchers didn’t understand the issues, fear of stigma/lack of anonymity, lack of trust in researchers for all the above), would anything have encouraged you to be more involved in research (probe: facilitators: better information (in plain English), trust-building exercises/inclusion of trusted people, training, clear and reasonable understanding of time/effort needed; financial reimbursement).   1. Do you use research when developing/communicating your services? (Probe: Anything you’ve looked for but not been able to find? |
| Give an overview of Research Partnership Network  This Research Partnership Network is being developed to provide an inclusive network of people involved in and affected by palliative and end of life care services. Its aim is to identify and ultimately address the major barriers to access and take up of services by ethnic minorities groups in Bedfordshire, Hertfordshire and Milton Keynes. These areas have large, diverse and influential ethnic communities but have previously been underrepresented/not represented in health-related research. This Research Partnership Network is being funded by the National Institute of Health Research (NIHR) to begin to address that imbalance, specifically for palliative and end of life care. The aim is to use partnerships to create a structure for the network that is truly inclusive and from this to identify the most important barriers to address. In the first instance this will lead to a published report and a submission for a substantial research project to address one of the most important issues identified.  Reasons for participation in research partnership network   - What influenced your decision to become involved in this research partnership network? (Probe: Importance of issue, impact of issue on daily activities, previously feeling unheard) - What’s important to you/will keep you engaged and involved within the research partnership network? - How important is connecting with other people/services involved in EoL care? - What will help you feel valued as part of this research network? - What would success of the network look like for you? |
|  |
| Representative structure   - What roles outside of your organisation are pivotal in people accessing your service(s)? (Probe: referral processes) - Looking at the structure (diagram provided) Is there anyone/any role you would like to see in the research partnership network that are not currently represented? |
| Research expectations   1. What would you like to see coming out of the research and how would you like to contribute?   Probe: skills they have might need; most important outcomes |
| Information/training /support   - What would help to facilitate research activity within your work? - What support would help you be more involved in this research activity? - What areas/skills would you like training in to help you feel more confident to engage in this research |

| Research topics questions |
| --- |
| Ethnic inequalities in accessing End-of-Life Care   - How do you currently engage with ethnic minority groups in your community? (Probe: successes, challenges; Which services? Who does not access the services?) - Are you aware of any other services/organisations that may have challenges in enabling ethnic minority groups to access their services? (Probe: similarities/differences from above) - Do you think these are the key challenges for ethnic minority groups in accessing end-of-life care services? Any others? (Probe, if not already mentioned: knowledge and awareness of services available to them? Understanding how to access a service? Perceptions and understanding of palliative care, religious/cultural beliefs and values, stigma, treatment preferences, language barriers, health literacy, assumptions that care will be provided by families, previous experience of health professionals) - Can you recommend any changes, based on your experience on how access to end-of-life care across minority ethnic populations could be improved? (Probe: Training; cultural competence) |
| Role of communication & translation   1. How important is communication in the access to and take up of your EoL services? 2. Conversations around palliative care and End of life can be difficult, even more so when there isn’t a shared culture and/or language. What do you see as the barriers and enablers to having such conversations effectively? (Probe: training; terminology) 3. How easy or difficult is it to translate written materials into the languages required? (probe: which languages are easy/difficult) 4. How easy or difficult is it find oral translators for the languages required? (probe: which languages are easy/difficult) 5. How can oral translation aid and hinder communication between a service provider and the user? 6. Do you think the person translating impacts on conversations – professionals, volunteer/faith, family? |
| Role of planning   1. How important do you think planning is for people in the last year of life? 2. Do people’s ethnic and/or faith backgrounds affect their attitudes towards planning for the end of life? (Probe: in what ways? Which groups?) 3. How well does an Advance Care Plan meet the needs of ethnic minority groups? (Probe: what’s good, what’s missing?) 4. How does advance care planning work within your organisation (Probe: training; ethnic differences; recording and sharing patient preferences) |
| Approaches to faith   1. How comfortable are you with the different faith approaches to death and dying? 2. Where do you get information from? 3. Do you get any training in the end of life requirements and issues of the major religions and cultures represented in your communities? 4. How does your understanding of different faiths affect the way in which you approach planning and services? 5. Does your organisation have access to a chaplaincy service? (Probe: experiences of access to suitable faith representatives) |
| Resources and administration   1. Do you have the resources to cope with current demand for your services? 2. What would happen if demand increased from ethnic groups not currently accessing services?   Impact of administrative boundaries   1. Do health authority, social services and county boundaries affect the way your services are delivered to ethnically diverse communities? (Probe: positives and challenges) |
| Next steps   1. What do you think should be the priorities for this research network in addressing inequalities in relation to access and uptake in End-of-Life Care? 2. Is there anything else you would like to share which I have not asked you? |
